# High-density LD-based structural variations analysis in ten Native and Mestizo Mexican populations

**DOI:** 10.1371/journal.pone.0333193

**Published:** 2025-09-25

**Authors:** Adriana Griselda Mateos-Valenzuela, Mirvana Elizabeth González-Macías, Carlos Villa-Angulo, Diana Helena Reyes-Godoy, Juan Carlos Fernandez-Lopez, Rafael Villa-Angulo

**Affiliations:** 1 Laboratory of Bioinformatics and Biophotonics, Engineering Institute, Autonomous University of Baja California, Mexicali, Baja California, México; 2 Faculty of Sports, Autonomous University of Baja California, Mexicali, Baja California, México; 3 National Insitute of Genomic Medicine (INMEGEN), Mexico City, Mexico; INNN: Instituto Nacional de Neurologia y Neurocirugia Manuel Velasco Suarez, MEXICO

## Abstract

The main objective of this study was to perform a genome-wide characterization of Structural Variations (SV) based on the deviation of the expected short-range Linkage Disequilibrium (LD) between Single Nucleotide Polymorphisms (SNPs) in 10 Native and Mestizo Mexican populations. We used a panel of 785,663 SNP genotypes, sampled from 383 individuals, of which 71 belonged to ethnic populations and 312 belonged to mestizo populations. The total number of variations found among all populations was 4,375, involving an average of 19,438 SNPs per population, which corresponds to the 3.14% of the total average of SNPs per population. The mean SV size varied from 2,845–8,646 kb across populations (with a mean SV size of 6,161 kb over all populations) and an average of 50.14 SNPs per SV. By grouping all variations across all populations in the sample we defined 506 regions, from which in 54 (11%) regions the 10 populations coincided. The total number of genes covered by these variations was 8,443. And, from all genes we identified some specifically related to Mexican health, as the genes *FTO* and *ABCA1* associated with obesity, with the adipose tissue function, and with the distribution of fat in Mexican population; the gene *ELMO1* associated with the susceptibility to diabetic nephropathy and diabetes type II, among others. In summary, our results add new evidence in support of the hypothesis that SVs based on the deviation of the expected short-range LD between SNPs capture the structure and the demographic history of populations, and represent potential targets for association of SVs with population-specific diseases.

## Introduction

The current Mexican population is mainly composed of two groups: Native and Mestizo. Native group corresponds to Indigenous Mexican communities that have remained largely unblended for centuries, owing to their cultural beliefs, and their geographic distance from urban settlements of the modern Mestizo population. Mestizo group corresponds to the admixture between native and foreign populations, which originated as a result of several historical events, including Spain’s conquest of America in 15th century, and African and Asian settlers arriving to Mexico.

Mexican Mestizo population has been a historical trend of increasing, becoming the dominant group. This growth is mainly attributed to intermarriage between indigenous women and European men, as a process that began soon after the arrival of the conquistadors. On the other hand, native groups faced a significant decline following the arrival of Europeans due to factors like social disruption and disease, being mainly affected by outbreaks of smallpox, measles, and typhoid disease between the years of 1521 and 1580 leading to a notable drop in their populations. Additionally, some Native communities predominantly settled in specific regions (center and southeast of the country), assuring the viability of water and rich soil for cultivating crops like corn, beans, and cacao; adapting and surviving to distinct environmental conditions [1,2].

The 2020 population census in México, conducted by the National Institute of Statistics and Geography (INEGI), showed that the total Mexican population in the year of 2020 were 126,014,026 individuals, with around 78% belonging to Mestizo population, 19.4% identified as Indigenous groups (Natives), 2% of Afro-descendant heritage, and 0.6% belonging to other groups [3].

Over the past decade, demographic studies in México, complemented with genetic information have revealed a high level of genetic diversity with an uneven distribution of alleles frequencies that differ according to the analyzed geographic region [4–7]. These studies demonstrated that the evolution of the Mexican population has been shaped by a complex interplay of historical, epidemiological, social, cultural, demographic, and economic events.

Even when Mexican population groups have been sharing territory, cultural traditions and language for centuries, notable genetic differences have been found within the same geographic regions, reflected in contrasting physical and genetic traits. Then, recent studies have been performed to analyze the genetic structure of Native and Mestizo Mexican populations; and to establish patterns in genetic variations of native ancestry or derived from admixture that could be associated with complex medical conditions, as well as physical and physiological characteristics. The analysis of genetic variability is helping to characterize the diversity in Mexican population and its impact on health. However, up to date, previous studies of Mexican genome diversity have mainly been focused in Single Nucleotide Variants (SNVs), small insertions and deletions (indels), Heterozygosity, and genetic differentiation based on Fixation index [2,4,6–8]. However, variations of other type rather than single genomic position have not been analyzed genome-wide. High density SNP markers evenly distributed in the genome enable the detection of regions with significant LD deviation compared to the expected value, which have been interpreted as short-range genomic variations, and could help in future studies for assessing association with other type of SVs [9]. In this work, we used a panel of 785,663 SNPs genome-wide, sampling 383 individuals from 3 Native and 7 Mestizo Mexican populations, provided by the National Institute of Genomic Medicine (INMEGEN). We used these data to inspect the distribution of structural variations based on the short-range LD patterns genome-wide for all the populations in the sample.

## Materials and methods

### Description of the data

The dataset analyzed in this study was provided by the Mexican government’s National Institute of Genomic Medicine, INMEGEN (http://inmegen.gob.mx). Data consisted of a panel of 785,663 SNP genotypes, sampled from 383 individuals belonging to 3 Native and 7 Mestizo Mexican populations. Populations and individuals were distributed as follow: Native populations were Maya (30 individuals), Tepehuano (20 individuals), and Zapoteca (21 individuals). While Mestizo populations were Guanajuato (48 individuals), Guerrero (50 individuals), Sonora (48 individuals), Tamaulipas (17 individuals), Veracruz (50 individuals), Yucatan (49 individuals), and Zacatecas (50 individuals). Taking in to account the geographic region in which populations are seatled we grouped Mestizos in two clusters: one cluster called Central-Coast Mestizo encompassing Tamaulipas, Guerrero and Veracruz populations; and another cluster called Non-Central-Coast Mestizo encompassing Guanajuato, Sonora, Zacatecas and Yucatan populations.

These data were previously analyzed in a research conducted and published by Silva-Zolezzi and Moreno-Estrada [4,8]. Genotype dataset is available under the term of a data transfer agreement to respect the privacy of the participants for the transfer of genetic data, by contacting INMEGEN (http://www.inmegen.gob.mx/). Written informed consent was obtained from all participants under the research/ethics approval number (2007/06) issued by the INMEGEN Ethics Commission. Data were accessed on 16/03/2022.

### Quality control filters

Quality Control filters were applied to data in order to guarantee a global quality over all the samples. All SNPs with Minor Allele Frequency (MAF) < 0.05 were removed. And, all SNPs that did not satisfy the Hardy-Weinberg equilibrium (P-value < 0.0001) were also removed. The initial number of SNPs was 7,856,630 from the 10 populations, and after filters, the finally number of SNPs was 6,195,350. It represents the 78.85% of the initial information.

### LD measure

The LD of every pair of SNPs in each chromosome, within each population, was estimated using the Pearson correlation formula (*r*^*2*^):


$$r^{\text{2}}=\frac{{\;(p_{\text{1}\text{1}}-p_{\text{1}}q_{\text{1}}\;\;\;\;)}^{\text{2}}}{\left(p_{\text{1}}q_{\text{1}}p_{\text{2}}q_{\text{2}}\right)}$$

Where *p*_*1*_ and *p*_*2*_ are the minor and major allele frequencies of SNP1 respectively, and *q*_*1*_ and *q*_*2*_ are the minor and major allele frequencies of SNP2 respectively. *p*_*11*_ corresponds to the frequency of observing both minor alleles in the same individual throughout the entire population. In addition, to avoid errors due to the sample size of each population, the following correction was applied to the LD:


$$r^{\text{2}}corrected=\frac{r^{\text{2}}computed-\frac{\text{1}}{n}}{\text{1}-\frac{\text{1}}{n}}$$

where *n* corresponds to the number of haplotypes in the sample [10], the *r*^2^ values were estimated with the PLINK tool [11].

### SV based on short-range LD

To estimate SVs across the entire genome, we implemented the definition of Short-Range LD-based SV made by Salomon, et al. [9]. It is defined as follows:

For each chromosome within each population, calculate the short-range (≤ 100Kb) LD (*r*^2^), sort the SNP pairs by distance and obtain a set of LD means (we called them expected means) using bins of 5Kb. Inspect all SNPs, from smallest to largest, looking for segments of at least 1 Kb, consisting of a set of at least 3 adjacent SNPs so that, for each SNP, *r*^2^ within its neighbors in a 100 Kb range, to the right side of that SNP, are all bigger than, or all smaller than, their corresponding expected means, and their *P*-values from a *t*-test for equality of means are significant after a Benjamini-Hochberg multiple testing correction. In addition, to account just for homogeneously distributed regions consider only SNPs having at least 15 SNP neighbors within a 100Kb range. Call these segments: High-Density SV based on short-range LD [9].

### Correction for multiple testing

A Benjamini-Hochberg correction for multiple testing [12] was applied to the set of *P-*values resulted from the application of the *t*-tests to each SNP with its neighbors in a range of 100 kb, in order to control the False Discovery Rate.The approach is as follows: first, all *P*-values are sorted from smallest to largest. Denote the *i*-th smallest *P*-value by *P*_*(i)*_, for each *i* between 1 and *m* (*m* is the total number of *P*-values), then, starting from the largest *P*-value *P*_(m)_, compare *P*_(m)_ with 0.5 x $\frac{i}{m}$. Continue as long as *P*_(i)_ > 0.5 x $\frac{i}{m}$. Let *k* be the first time when *P*_(k)_ is less than or equal to 0.5 x $\frac{k}{m}$, and declare the differences corresponding to the smallest k *P*-values as significant.

### Principal components analysis (PCA)

Using the number SVs found for each chromosome, in each population, vectors of 22 dimensions were generated and a PCA was applied looking for differentiation between populations [13]. PCA was performed using the R software [14].

## Results

### MAF distribution

**Fig 1:**
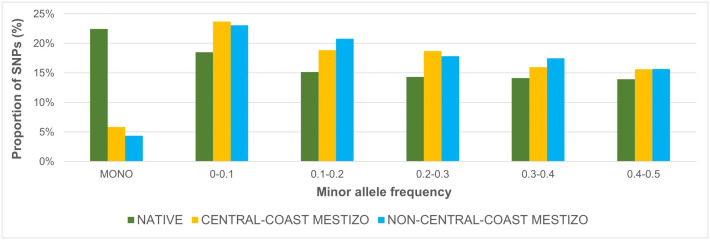
MAF distribution. Average proportions of SNPs of various frequencies by population group (including the intervals´ upper limit).

To investigate how informative the SNPs sampled in the 10 populations were, we computed the distribution of MAF across all chromosomes and all populations. Fig 1 shows the averaged proportions grouping populations as Native (Zapoteca, Tepehuano, Maya), Central-Coast Mestizo (Tamaulipas, Guerrero, Veracruz), and Non-Central-Coast Mestizo (Guanajuato, Sonora, Zacatecas, Yucatan). Native group presented notably more monomorphic SNPs (~23%) than Mestizo groups (~5%). While Mestizo groups presented consistently bigger polymorphic proportions in the rest of the bins.

S1 File shows MAF proportions from each population. As we can see, the 3 Native populations presented consistently lower polymorphic proportions (between 71% and 83%), compared to Mestizo populations (between 88% and 96%). However, a substantial fraction of loci are informative in all study populations.

The MAF values vary from a maximum of 0.227 (Sonora) to 0.177 (Zapoteca), which is a difference of 10% in the complete scale of 0.0 to 0.5. The average MAF decrease between populations is 1.1% (S2 File). Monomorphic SNPs were excluded from the following analyses.

### Extent of LD

**Fig 2:**
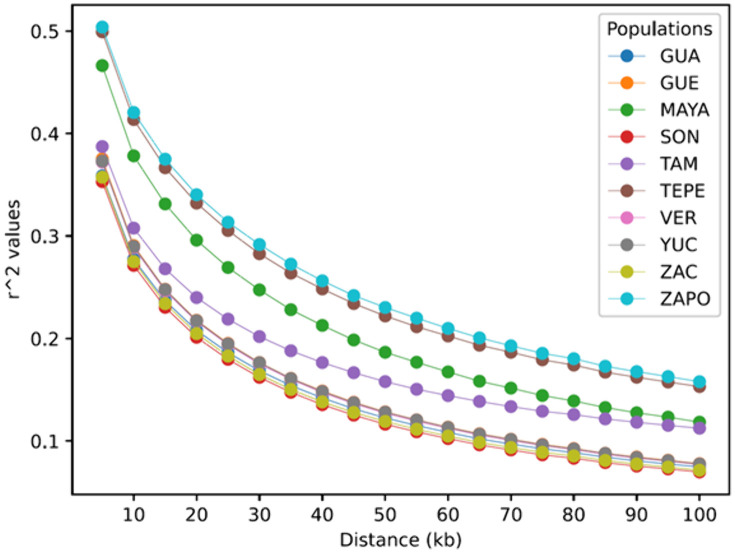
LD. Genome-wide LD decay in all populations.

The average number of SNPs per population after quality control filters was 619,535. Therefore, they were used to evaluate the extent of LD in a range of 100 kb. The pairwise LD correlation coefficient *r*^2^ was computed for every pair of SNPs within a range or 100 kb in each chromosome, within each population. Fig 2 shows the average of *r*^2^ values using 5 kb bins. As we can see, the decline in LD as a function of distance is rapid, such that *r*^2^ averages ~0.10 over 100 kb. Native populations show uniformly higher LD values relative to other populations. In shorter distances (5 kb), Zapoteca, Tepehuano, and Maya show higher *r*^2^ values (~0.49 on average), while the rest of the populations show an average of ~0.37. In longer distances (100 kb), Zapoteca and Tepehuano show consistently higher values, with an average of ~0.156. The populations of Guanajuato, Guerrero, Sonora, Veracruz, Yucatan, and Zacatecas show the lowest values, with an average of 0.075. While the Maya and Tamaulipas populations show intermediate values, with an average of 0.115 (S3 File).

### Estimation of SVs based on short-range LD

SV based on short-range LD were estimated using the definition from [9]. The algorithm is described in Materials and methods section. Table 1 details the SV characteristics for all populations. In summary, the total number SVs found among the 10 populations was 4,375, involving an average of 19,437.6 SNPs per population, which corresponds to the 3.14% of the total average of SNPs per population. We found that SV mean size varied from 2,844.93 to 8,646.07 kb across populations (with a mean SV size of 6,161.1 kb over all populations) and an average of 50.14 SNPs per SV.

**Table 1:** SV statistics genome-wide across all populations.

*Population*	*No of SVs*	*Total of SNPs*	*SNPs per SV (Max)*	*SNPs per SV (Average)*	*SVs per chromosome (Average)*	*Total size (Mb)*	*Max SV size (Mb)*	*Min SV size (Kb)*	*SV size average (Kb)*	*SV mean size 95% Confidence Interval (min, max) in kb*	
***GUANAJUATO***	325	21805	694	67.09	14.77	2487.0	96.2	3.37	7652.18	6502.98	8801.37
***GUERRERO***	351	22955	475	65.4	15.95	2468.7	57.1	1.48	7033.33	6086.68	7979.99
***SONORA***	362	23086	654	63.77	16.45	2509.6	65.7	1.73	6932.49	5981.81	7883.17
***TAMAULIPAS***	288	10135	251	35.19	13.09	2490.1	82.4	5.38	8646.07	7417.59	9874.56
***VERACRUZ***	354	23359	634	65.99	16.09	2487.3	80.1	1.56	7026.24	6047.85	8004.64
***YUCATAN***	363	23082	470	63.59	16.5	2519.1	49.6	1.24	6939.66	6058.78	7820.54
***ZACATECAS***	342	23503	507	68.72	15.55	2515.4	59.0	1.41	7355.1	6386.89	8323.31
***MAYA***	582	18602	424	31.96	26.45	2275.6	50.7	1.18	3909.9	3436.35	4383.46
***TEPEHUANO***	678	14639	200	21.59	30.82	2217.9	50.5	1.34	3271.17	2875.86	3666.47
***ZAPOTECO***	730	13210	154	18.1	33.18	2076.8	36.6	1.12	2844.93	2533.07	3156.79

The Maya, Tepehuano and Zapoteca populations showed the largest number of SVs, with 582, 678 and 730 respectively, while Tamaulipas, Guanajuato and Zacatecas showed the smallest number, with 288, 325 and 342 SVs, respectively. The average distance covered by SVs genome-wide was 2.4 Mb. The biggest SV was found in Guanajuato population, with 96.2 Mb size, while the smallest SV was found in Zapoteca population, with 1.12 kb size.

The average number of SVs per chromosome was 19.93. The chromosome with the highest average of SVs was chromosome 2, with 37.6 SVs, while the chromosome with the smallest average of SVs was chromosome 19, with 4 SVs.

**Fig 3:**
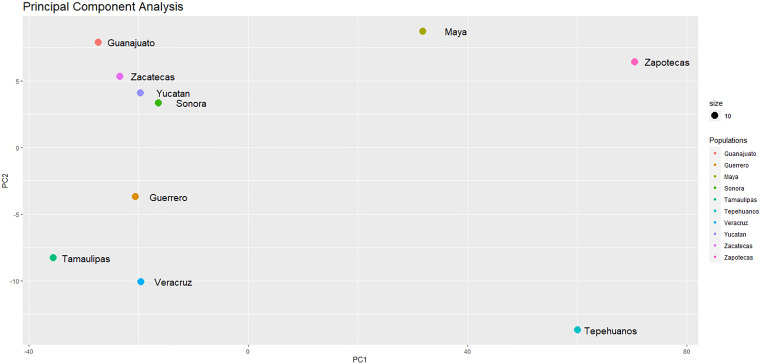
PCA plot. PCA on SVs per chromosome vectors shows a clear differentiation between population groups.

In order to investigate the closeness in variability among populations, given the number of identified SVs; for each population we constructed a vector of 22 fields, where each field contained the number of SVs in a chromosome (S4 Table). PCA [13] was applied to these vectors. Fig 3 shows a plot of PC1 vs PC2.

As we can see, the plot of PC1 vs PC2 shows a clear differentiation, based on the number of SVs per chromosome genome-wide, between the groups we investigated. For PC1, the three Native populations have positive loadings, while all Mestizo populations have negative loadings. Actually, the three main subgroups are clearly differentiated. In the first quadrant of the plot (PC1 and PC2 positive loadings) appear Maya and Zapoteca populations. In the second quadrant (PC1 negative and PC2 positive loadings) appears the Non-Central-Coast Mestizo subgroup. In the third quadrant (PC1 and PC2 negative loadings) appears the Central-Coast Mestizo subgroup. And, in the fourth quadrant (PC1 positive and PC2 negative loadings) appears Tepehuano population. This result supports the hypothesis stated previously [6] that demographic and adaptive processes that occurred in these groups shaped their genetic architecture.

In order to identify genes involved in our defined SVs, we did an inspection in NCBI (https://www.ncbi.nlm.nih.gov/) looking for genes overlapping the defined SVs. For each defined SV in each chromosome, across all populations, we investigated if a gene was overlapping the SV; and for each overlapping gene we investigated if there were more SVs in the rest of populations, overlapped by the same gene. S5 Table presents for each chromosome, the genes, the start and end position of each gene, the gene ID, the gene function, and the populations with SVs covered by the gene. In total, we identified 8,443 genes covering SVs across all populations. From this total, 790 genes contained SVs in all populations; 2,149 genes contained SVs in only one of the populations, and 5,504 genes contained SVs in more than one but less than ten populations. When inspecting by group, we found that 26 genes contain SVs across all Mestizo populations; while 14 genes contained SVs across all Native populations.

The last analysis was to look within our defined SVs for regions that were consistent between populations. We found 506 regions, from which in 54 (11%) regions the 10 populations coincided, while in 90 (18%) regions just 1 population coincided (S6 Table).

## Discussion

In this work, we implemented an intuitive and simple definition of SV based on the deviation of the expected short-range LD between SNPs, recently introduced by Salomon, et al., [9]. In his work, Salomon studied SVs in the cattle genome, and concluded that the short-range LD patterns captured by these SVs resume enough genetic information to discern relatedness of breeds given the geographic regions in which they are evolving. In this work, we studied SVs in 10 Native and Mestizo human Mexican populations, and we concluded that our results add new evidence in support of the same conclusion. SVs inferred from our data showed a good populations differentiation when applied to clustering analysis, suggesting that they are defined by the population structure and the demographic history, as Ávila et al. 2020 [5] has previously stated.

From the MAF analysis, the three Native populations presented consistently lower polymorphic proportions (between 71% and 83%), compared to Mestizo populations (between 88% and 96%). And, within Natives, Zapotecas population resulted with the highest proportion of monomorphic SNPs, while at the same time showed consistently bigger LD than the rest populations in the range of 100kb, and presented the highest number of SVs genome-wide. This result can be explained due to the fact that Zapoteca has been historically the most isolated people due to geographic and cultural barriers. Then, is the ethnic group with the lowest genetic exchange.

The total number of SVs found in our study was 4,375 which is a much lower quantity than those reported in previous studies of variations in Native and Mestizo Mexican populations. The reason of this difference is that previous studies have mainly focused on SV variants type, while in our study we focused on short-range LD genomic variations defined by regions that consist of at least three adjacent SNPs that present significant LD deviation compared to the expected value. Avila et al., Romero et al., and Aguilar et al. [5–7], for example, reported 120,735, 332,272 and 8.68 million of the SNV type, in Native and Indigenous Mexican populations, respectively. The difference in the amount of our SVs compared to the SNVs found in previous studies is in accordance with Chiang et al., 2017 [15], who mentions that approximately 5,000–10,000 SVs can be found in the human genome, since they are less abundant compared to SNVs, which can exceed the 4 million.

PCA analysis defined a strong axis of variation separating Native from Mestizo populations when observed from PC1. But when observed from both PC1 and PC2, two clusters are clearly visible. One of the clusters is formed by Tamaulipas, Guerrero and Veracruz populations, all situated in the central coast of Mexico, which is an area with a rich and complex history. Before the arrival of the European conquerors the central coast was the home of diverse native ethnic groups, such as Huastecos, Otomies, Nahuas, Totonacos, and Mixtecos. After the conquest and the beginning of the miscegenation all these ethnic groups saw their population decrease dramatically. The other cluster is formed by Guanajuato, Zacatecas, Yucatan and Sonora populations, all situated out of the central coast area, and with a slightly different history after the conquest, since miscegenation started mainly in the central coast area and spreaded out to the rest of Mexico. This PCA result reflect the role that the geographic location, the degree of isolation, and the interbreeding within Native and between Native and foreign populations have played in the actual conformation of the modern Mexican population diversity, and are in agreement with the study published by Sohail, et al., 2023 [16], who analyzed the genetic and environmental factors of actual Mexican populations, and reported that ancestry differences in Mexican population are present mainly in the center and south of the country, which is the area with the highest american ancestry; besides genetic flow found in the Atlantic-coastal corridor, determining the genetic influence between the center-south and south-east Mexican populations [16].

On their side, the three Native populations appear separated when observed from both PC1 and PC2. At the top right side of the plot in Fig 3, with loadings between 60 and 80 for PC1 and between 5 and 7.5 for PC2, appears Zapoteca population, while in the bottom right side, with loadings of 60 for PC1 and between 12.5 and 15 for PC2, appears Tepehuano population. And, close to top center with loadings between 0 and 40 for PC1 and between 7.5 And 10 for PC2, appears Maya population. Assuming this separation of Native populations as an indicative of genetic differences between Native populations due to population structure and demographic history, then our result is in agreement with the result reported by Moreno-Estrada [4], who analyzed SNVs in 20 Mexican Indigenous groups and reported that they differentiate in three main areas according to its geographic location: the northerns considering the Tepehuano, the southerns considering Zapoteca and Maya. In addition, Romero et al., [6], reported that Native groups are formed by three main ancestral components: a northern, a southern and a Mayan component; where Tepehuano population shows the highest proportion values of the northern component, the Zapoteca population shows highest proportion values of the southern component, and the Maya population shows their own component. This separation was reported too, across all original inhabitants of the American continent population (generally called Native Americans) by Raghavan et al., [17] who, using ancient and modern genome-wide data found that the ancestors of all present-day Native Americans entered the Amercias as a single migration wave from Siberia no earlier than 23 thousand years ago, and from that migration, there was a diversification of ancestral Native Americans leading to the formation of northern and southern branches. Other anthropological studies realized by González et al., [18] indicated that the first human vestiges in México were presented approximately 12,000 years ago, in the center zone of Mexico, and the peninsula of Yucatán.

Next, we did an inspection in NCBI looking for genes involved in our defined SVs. In total we identified 8,443 genes covering SVs across all populations. From this total, 790 genes contained SVs in all populations; 2,149 genes contained SVs in only one of the populations, and 5,504 genes contained SVs in more than one but less than ten populations. When inspecting by group, we found that 26 genes contain SVs across all Metizo populations; while 14 genes contained SVs across all Native populations. In addition to the genes reported by other studies [7,19], we identified genes like *FTO* and *ABCA1* in the 10 populations. These genes have been related to obesity, to the adipose tissue function, and to the distribution of the fat in mexican population [20,21]. Another gene was *ELMO1*, that is associated with the susceptibility to diabetic nephropathy and diabetes type II, as documented by Aguilar et al., [7]. In addition, we identified the gene *BRCA2* in the Guerrero, Maya, and Zapoteca populations, and the gene *IKBKB* in Guanajuato, Sonora, Tamaulipas, Tepehuano and Yucatan populations. These genes were previously reported by Moreno et al., [4], and Aguilar et al. [7], as associated to the development of breast and ovarian cancer in the Mexican population. Additionaly, we found genes located in only one of the populations, like the *MCHR1*, implicated in the neural regulation of food consumption, found only in Zacatecas population. The gene *SLC30A8*, which confers certain disposition to non-insulin-dependent diabetes, found only in the Guanajuato population. And, the gene *IGF2 BP2*, which plays an important role with metabolism associated to the susceptibility of diabetes, found only in the Tamaulipas population.

## Conclusion

We present the first genome-wide characterization of SVs based on the deviation of the expected short-range LD between SNPs in Native and Mestizo Mexican populations. The total number of variations found among all populations was 4,375, involving an average of 19,438 SNPs per population, which corresponds to the 3.14% of the total average of SNPs per population. The mean SV size varied from 2,845–8,646 kb across populations (with a mean SV size of 6,161 kb over all populations) and an average of 50.14 SNPs per SV. By grouping all variations across all populations in the sample we defined 506 regions, from which in 54 (11%) regions the 10 populations coincided. The total number of genes covered by these variations was 8,443. And, from all genes we identified some specifically related to Mexican health, as the genes *FTO* and *ABCA1* associated with obesity, with the adipose tissue function, and with the distribution of fat in Mexican population; the gene *ELMO1* associated with the susceptibility to diabetic nephropathy and diabetes type II, among others. Finally, our results add new evidence in support of the hypothesis that SVs based on the deviation of the expected short-range LD between SNPs capture the structure and the demographic history of populations, and represent potential targets for association of SVs with population-specific diseases. In further analyses, the inclusion of phenotypic data would be necessary in order to establish association of SVs with physical traits or disease-related outcomes.

## Supporting information

S1_File MAF Distribution and Minor Allele Frequency distribution. (PDF)

S2_File Average minor allele frequencies (MAF) per population in the study. (PDF)

S3_File Total average of *r*^*2*^ per population and LD decay using 5 kb bins. (PDF)

S4_Table Table of Number of SVs per chromosome in each population. (XLSX)

S5_Table Table of Genes involved in Structural Variations. (XLSX)

S6_Table Table of Structural Variaton Regions. (XLSX)
