# Supplementary material for: High-density LD-based structural variations analysis in ten Native and Mestizo Mexican populations
Source: PLoS One. 2025 Sep 25;20(9):e0333193. doi: 10.1371/journal.pone.0333193 (PMC12463268; doi:10.1371/journal.pone.0333193)
Supplement: S1_File — (PDF) [file pone.0333193.s001.pdf]

Supporting Information 1

MAF Distribution and Minor Allele Frequency distribution

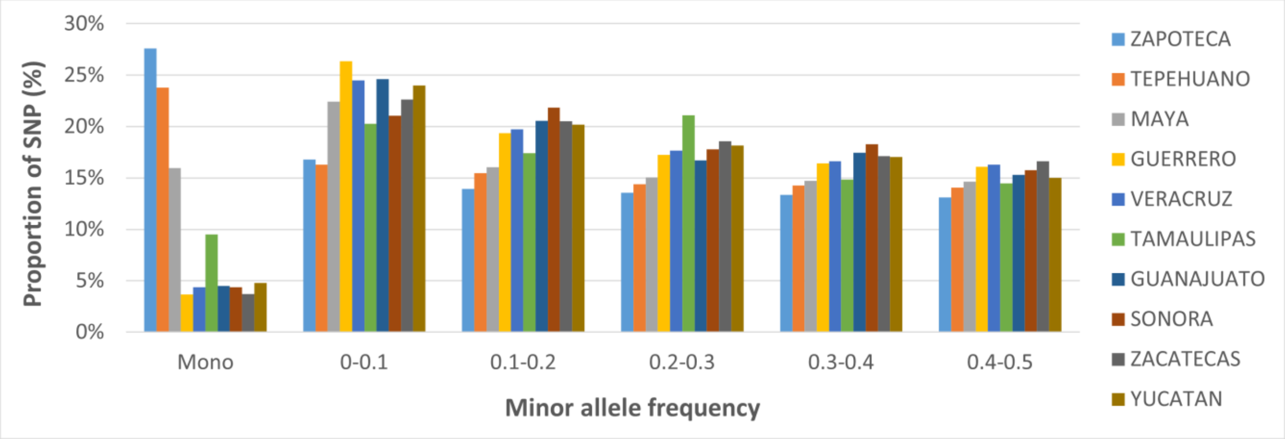

Average proportions of SNPs of various frequencies by population (intervals' upper limit inclusive).

Minor Allele Frequency Table

	Mono	0-0.1	0.1-0.2	0.2-0.3	0.3-0.4	0.4-0.5	Polymorphic Proportion (%)
ZAPOTECA	0.276	0.168	0.139	0.136	0.134	0.131	70.76
TEPEHUANO	0.238	0.163	0.155	0.144	0.143	0.141	74.47
MAYA	0.160	0.224	0.160	0.151	0.147	0.146	82.87
GUERRERO	0.037	0.263	0.194	0.173	0.164	0.161	95.50
VERACRUZ	0.044	0.245	0.197	0.177	0.166	0.163	94.82
TAMAULIPAS	0.095	0.203	0.174	0.211	0.148	0.145	88.12
GUANAJUATO	0.045	0.246	0.205	0.167	0.175	0.153	94.65
SONORA	0.044	0.211	0.218	0.178	0.183	0.158	94.75
ZACATECAS	0.037	0.226	0.205	0.186	0.171	0.166	95.49
YUCATAN	0.048	0.240	0.202	0.181	0.170	0.150	94.39
