# Supplementary material for: High-density LD-based structural variations analysis in ten Native and Mestizo Mexican populations
Source: PLoS One. 2025 Sep 25;20(9):e0333193. doi: 10.1371/journal.pone.0333193 (PMC12463268; doi:10.1371/journal.pone.0333193)
Supplement: S2_File — (PDF) [file pone.0333193.s002.pdf]

Supporting Information 2

Average minor allele frequencies (MAF) per population in the study

Population	Average MAF	Value in the scale 0.0 – 0.5 (%)	Decay with respect to the previous population (%)
Sonora (Mestizo non-coastal area)	0.227	45.4	0
Zacatecas (Mestizo non-coastal area)	0.223	44.6	0.8
Guanajuato (Mestizo non-coastal area)	0.218	43.6	1
Yucatan (Mestizo non-coastal area)	0.218	43.6	0
Tamaulipas (Mestizo coastal area)	0.218	43.6	0
Veracruz (Mestizo coastal area)	0.217	43.4	0.2
Guerrero (Mestizo coastal area)	0.214	42.8	0.6
Maya (Native)	0.189	37.8	5
Tepehuano (Native)	0.181	36.2	1.6
Zapoteca (Native)	0.177	35.4	0.8
