# Supplementary material for: High-density LD-based structural variations analysis in ten Native and Mestizo Mexican populations
Source: PLoS One. 2025 Sep 25;20(9):e0333193. doi: 10.1371/journal.pone.0333193 (PMC12463268; doi:10.1371/journal.pone.0333193)
Supplement: S3_File — (PDF) [file pone.0333193.s003.pdf]

Supporting Information 3

Total average of r^2 per population and LD decay using 5 kb bins

Population	r^2 average
Guanajuato	0.145
Guerrero	0.152
Sonora	0.139
Tamaulipas	0.180
Veracruz	0.151
Yucatan	0.152
Zacatecas	0.142
Maya	0.213
Tepehuano	0.248
Zapoteca	0.255
Total r^2 average	0.178

LD decay using 5 kb bins

POPULATION	5K	10K	15K	20K	25K	30K	35K	40K	45K	50K	55K	60K	65K	70K	75K	80K	85K	90K	95K	100K	AVERAGE
GUA	0.359	0.277	0.237	0.208	0.186	0.169	0.154	0.142	0.131	0.123	0.115	0.108	0.102	0.097	0.092	0.089	0.084	0.081	0.078	0.075	0.145
GUE	0.375	0.291	0.248	0.218	0.195	0.177	0.161	0.149	0.138	0.128	0.121	0.114	0.107	0.102	0.097	0.093	0.088	0.084	0.081	0.078	0.152
SON	0.353	0.271	0.230	0.201	0.180	0.162	0.148	0.135	0.125	0.116	0.109	0.102	0.096	0.091	0.086	0.083	0.079	0.075	0.073	0.070	0.139
TAM	0.387	0.308	0.268	0.240	0.219	0.202	0.188	0.177	0.167	0.158	0.150	0.144	0.139	0.134	0.129	0.126	0.122	0.118	0.115	0.112	0.180
VER	0.372	0.289	0.247	0.217	0.194	0.175	0.160	0.147	0.136	0.127	0.119	0.112	0.106	0.101	0.096	0.092	0.087	0.083	0.080	0.077	0.151
YUC	0.373	0.290	0.248	0.217	0.195	0.176	0.161	0.148	0.137	0.128	0.120	0.113	0.107	0.101	0.096	0.092	0.088	0.084	0.081	0.078	0.152
ZAC	0.358	0.275	0.234	0.205	0.183	0.165	0.150	0.138	0.128	0.119	0.111	0.105	0.099	0.094	0.089	0.085	0.081	0.078	0.075	0.071	0.142
MAYA	0.466	0.378	0.331	0.296	0.269	0.247	0.228	0.213	0.199	0.187	0.177	0.167	0.158	0.152	0.144	0.139	0.133	0.128	0.123	0.118	0.213
TEPE	0.499	0.414	0.367	0.332	0.305	0.283	0.264	0.248	0.234	0.222	0.212	0.202	0.193	0.187	0.179	0.174	0.167	0.162	0.158	0.153	0.248
ZAPO	0.504	0.421	0.375	0.340	0.314	0.292	0.273	0.256	0.242	0.230	0.220	0.210	0.201	0.193	0.186	0.180	0.173	0.168	0.163	0.158	0.255
